# The Ross Procedure in Children with Congenital Heart Disease

**DOI:** 10.3390/jcdd12050186

**Published:** 2025-05-15

**Authors:** Nabil Dib, Nancy Poirier, Ismail Bouhout, Paul Khairy

**Affiliations:** 1 Department of Medicine, Montreal Heart Institute, Université de Montréal, Montreal, QC H1T 1C8, Canada; nabil.dib@anemf.org; 2 Division of Cardiac Surgery, Department of Surgery, Haut Leveque Hospital, Université de Bordeaux, 33000 Bordeaux, France; 3 Division of Cardiac Surgery, Department of Surgery, Sainte-Justine Hospital, Université de Montréal, Montreal, QC H3T 1C5, Canada

**Keywords:** aortic valve disease, Ross procedure, congenital heart disease, pediatric cardiology

## Abstract

Aortic valve disease accounts for approximately 5% of all congenital heart defects in children. Choosing the optimal valve replacement in this population is challenging, as it must ensure durability, accommodate growth, and minimize the need for long-term anticoagulation. Biological valves do not require anticoagulation but lack durability and growth potential, leading to frequent reoperations. Mechanical valves offer longevity but necessitate lifelong anticoagulation and do not grow with the child. Among the available surgical options, the Ross procedure has emerged as a preferred approach due to its favorable hemodynamic performance, growth potential, and freedom from anticoagulation. First described in 1967, this technique involves replacing the diseased aortic valve with a pulmonary autograft and reconstructing the right ventricular outflow tract using a human or non-human valve substitute. Despite its advantages, the procedure is technically demanding, has a considerable learning curve, and transforms a single-valve pathology into a bivalvular condition. This narrative review provides an updated perspective on the Ross procedure in children, focusing on long-term survival, reoperation rates, and the role of percutaneous valve replacement in delaying surgical reintervention. By synthesizing the latest evidence, we aim to clarify the current standing of the Ross procedure as a durable and effective solution for pediatric aortic valve disease.

## 1. Introduction

The Ross procedure, introduced by Donald Ross in 1967, is a surgical technique for treating aortic valve disease by replacing the diseased aortic valve with a pulmonary autograft [1]. Among children with congenital heart disease (CHD), aortic valve disease accounts for approximately 5% of cases, ranking it among the most common forms of CHD [2,3]. Identifying an ideal aortic valve substitute in pediatric patients is challenging, as it must balance durability and growth potential, while minimizing thromboembolic risk without requiring long-term anticoagulation. Biological valves eliminate the need for anticoagulation but have limited durability, in part due to their inability to grow with the child. As a result, nearly half of patients implanted before the age of 35 years require reoperation within 10 years [4]. Mechanical valves, while durable, necessitate lifelong anticoagulation and do not accommodate somatic growth, posing additional long-term management challenges.

The Ross procedure addresses these limitations, offering growth potential, freedom from anticoagulation, and favorable hemodynamics [5,6]. However, it has major drawbacks. It is a technically demanding procedure with a steep learning curve, requiring surgeons to perform an estimated 75–100 cases to achieve proficiency [7]. Additionally, it converts a single-valve disease into a bivalvular condition, potentially leading to future complications in both the autograft and the right ventricular outflow tract (RVOT). This narrative review provides an updated perspective on the Ross procedure in children, focusing on long-term survival and reoperations, along with evolving strategies to improve outcomes.

## 2. Evolution of the Ross Surgical Technique

The original description of the Ross procedure employed a subcoronary technique, which consisted of seating the pulmonary autograft within the aortic root beneath the coronary ostia [1]. One of the key challenges of this technique is achieving a precise geometrical match between the pulmonary autograft and the native aortic root. Since the pulmonary valve has a different shape and leaflet geometry compared to the native aortic valve, surgical adjustments are necessary to optimize valve function. Any asymmetry or distortion can lead to regurgitation or early valve failure. Consequently, this technique can be technically demanding, contributing to variable outcomes. Due to these challenges, the full root replacement variant has emerged as the preferred approach. Preserving the cylindrical geometry of the pulmonary root enhances valve competence, leading to improved long-term function and durability [8]. However, one significant drawback of the full root technique is the risk of coronary artery distortion after implantation, which can compromise coronary perfusion and lead to ischemic complications. Beyond these technical challenges, the pulmonary autograft itself presents unique concerns due to its intrinsic fragility. This necessitates meticulous suturing and careful postoperative blood pressure management to minimize complications.

Despite initial success, the widespread adoption of the Ross procedure slowed after reports of neoaortic root dilation in the second postoperative decade, which, in some cases, progressed to aortic dissection and autograft failure [9]. To address this complication, the inclusion technique was proposed, in which the pulmonary autograft is supported within an external reinforcement. Various levels of reinforcement have been explored, including annular support, stabilization at the sino-tubular junction, and reinforcement around the sinuses. However, the optimal approach remains a subject of ongoing debate, particularly in adults [10,11]. In young children, annular fixation is generally avoided to preserve the growth potential of the autograft [12].

## 3. Survival Following the Ross Procedure

Advancements in surgical techniques and perioperative care have significantly reduced postoperative mortality in children undergoing the Ross procedure [13]. However, early mortality rates remain highly variable, with a systematic review reporting figures ranging from 0.0% to 17.0%, largely influenced by patient-specific factors, particularly age at the time of surgery [14]. Neonates (<30 days old) are at the highest risk, as demonstrated in another recent meta-analysis, which found early mortality rates as high as 24% and late mortality rates reaching 43% [15]. This risk is further exacerbated in low-volume centers, where outcomes tend to be inferior compared to high-volume institutions [16,17]. These findings align with earlier smaller studies, which also reported high in-hospital mortality rates in neonates [18,19]. In contrast, large, high-performing centers have reported negligible mortality, highlighting the importance of surgical experience and institutional volume in determining outcomes.

**Figure 1:**
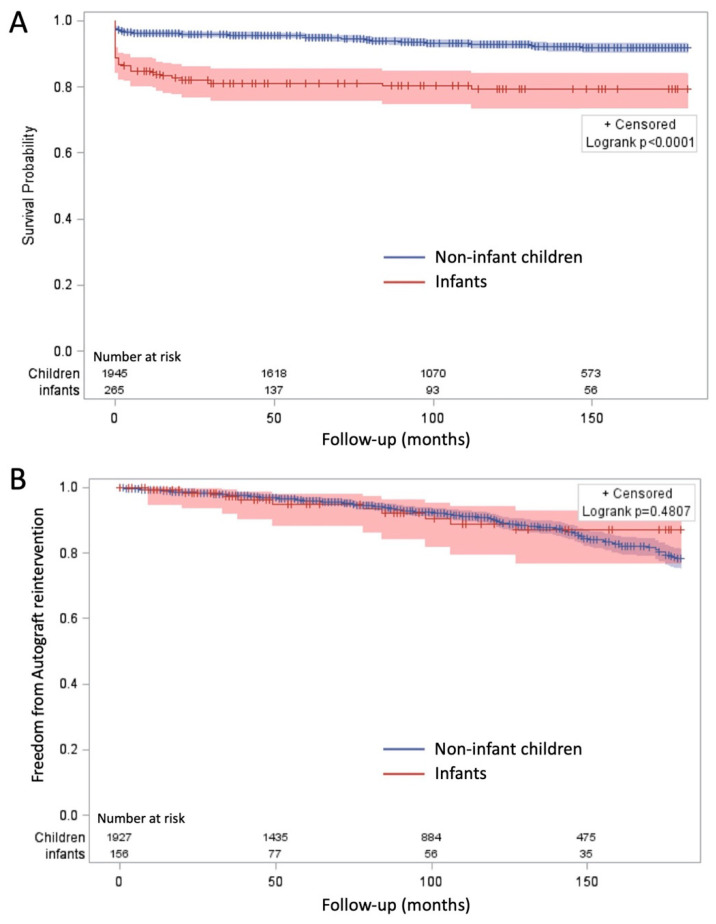

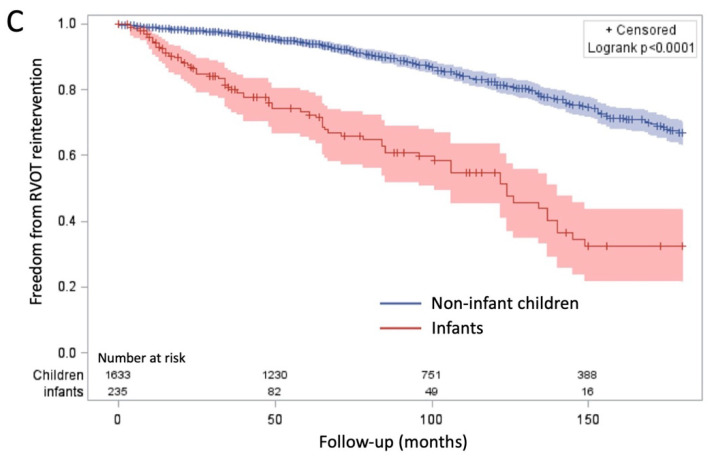
Survival (**A**), freedom from pulmonary autograft reintervention (**B**), and freedom from right ventricular outflow tract reintervention (RVOT); (**C**) after the Ross procedure in children an in the subgroup of infants. Reproduced with permission from Dib N et al. [20].

A recent meta-analysis using a time-to-event methodology attempted to provide a more accurate estimation of long-term survival by minimizing the bias introduced by heterogeneous follow-up durations [20]. By extracting individual patient data and digitalizing Kaplan–Meier survival curves, the estimated 15-year survival after the Ross procedure was 90.1% (Figure 1A). Notably, mortality among children undergoing the Ross procedure was significantly lower than that observed in cohorts receiving aortic valve replacement (AVR) with either a bioprosthetic [21] or mechanical valve [22,23]. Furthermore, multiple studies have shown that the long-term survival of Ross-operated patients closely approximates that of the general population [24], with life expectancy estimated at 90–95% of that of age- and sex-matched peers. Importantly, research suggests that patients who have undergone the Ross procedure experience no significant impairment in quality of life or restrictions in daily activities [11,25], reinforcing its durability and physiological advantages over alternative valve replacement options.

## 4. Reoperations After the Ross Procedure

Although the Ross procedure provides excellent survival outcomes in children, reoperation remains a consideration due to structural valve deterioration affecting both the pulmonary autograft and the RVOT conduit. The durability of the autograft is largely attributed to its ability to grow with the patient, but long-term function may be compromised by progressive dilation and valvular dysfunction.

### 4.1. Autograft Deterioration and Reintervention

Reintervention rates for the autograft vary substantially across studies, ranging from 0.37% to 2.81% per year [14], with a recent meta-analysis (Figure 1B) estimating freedom from autograft reoperation at 90% at 10 years [20]. The primary mechanism of failure is autograft regurgitation, which typically results from dilation of the neoaortic root [26,27]. Several factors contribute to this progressive dilation, including the intrinsic properties of the pulmonary artery wall, which contains less elastic fiber density than the native aortic wall [28]. This structural difference makes the pulmonary valve more susceptible to mechanical stress and systemic arterial pressure, increasing the likelihood of dilation over time [29]. The risk is particularly high in patients with preoperative aortic insufficiency or annular dilation, where the native support structures are already compromised.

In an effort to prevent early dilation, strict blood pressure control in the first six postoperative weeks has been recommended by Yacoub et al. [30]. Additionally, several surgical modifications have been proposed to reinforce the autograft, including external support with a Dacron tube, root annuloplasty, or incorporation of the autograft within the native aortic root using the subcoronary technique. However, the efficacy of these techniques depends largely on surgeon experience and institutional expertise. Furthermore, while reinforcement strategies may improve durability in adults, they are generally avoided in young children to preserve the natural growth potential of the autograft [31,32].

When reintervention on the autograft is required, the preferred approach is aortic valve-sparing surgery, which allows preservation of the native autograft whenever possible [33]. Unlike survival outcomes, which demonstrate a clear advantage of the Ross procedure over non-Ross AVR, reintervention rates are less consistent across studies. Some reports suggest a higher risk of late reoperation following the Ross procedure, particularly in patients with rheumatic aortic valve disease, a condition associated with poorer long-term outcomes [22]. In contrast, research by Sharabiani et al. found that reintervention rates at 10 years were lower in Ross-operated patients compared to those with mechanical AVR, likely due to the lack of patient-prosthesis mismatch, which can occur in growing children with fixed prosthetic valves [21].

A unique aspect of the Ross procedure is that it creates a bivalvular condition, meaning that both the autograft and the RVOT conduit may eventually require intervention [34]. This distinguishes it from other forms of AVR, where the need for reoperation is limited to a single valve.

### 4.2. RVOT Reconstruction and Reintervention

**Figure 2:**
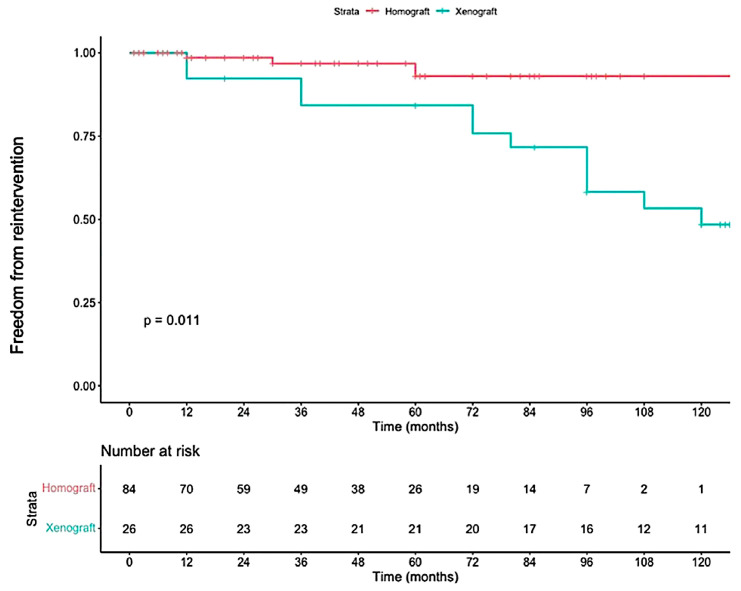
Freedom from reintervention on the right ventricular outflow tract according to whether a pulmonary homograft or xenograft (Freestyle or Contegra) was implanted. Reproduced with permission from Dib N et al. [35].

Reconstruction of the RVOT is an important aspect of the Ross procedure. The type of conduit used often depends on regional availability and institutional preference. In North America, pulmonary homografts are the most frequently implanted, while in other regions where homografts are less accessible, xenografts (such as bovine jugular vein conduits) are more commonly used. However, as shown in Figure 2, xenografts have been associated with inferior durability compared to homografts, leading to earlier reoperation [35].

Unlike autografts, which typically fail due to regurgitation and dilation, RVOT conduits primarily fail due to stenosis. Several factors influence the likelihood of conduit deterioration, including age at implantation, immune-mediated degeneration, and surgical expertise. Younger patients, particularly infants under one year of age, experience higher conduit failure rates (Figure 1C), as non-living conduits do not grow, resulting in progressive RVOT obstruction over time [20,21]. Additionally, the immune system plays a role in accelerated conduit degeneration, particularly in xenografts, which may undergo rapid calcification [36].

A recent meta-analysis reported that RVOT reoperation rates range from 0.3% to 4.8% per year, with younger children experiencing higher rates of reintervention (4.2–6.6% per year) compared to older children (0.3–4.8% per year) [14]. The progressive nature of patient-prosthesis mismatch in young children is a major factor in these higher reoperation rates. In an effort to delay reintervention, some surgeons advocate oversizing the homograft at the time of implantation, although this strategy has variable success [37]. To address immune-mediated degeneration, decellularized homografts have been developed to reduce the immune response and enhance graft longevity by allowing colonization with the recipient’s own cells [38,39]. Early results suggest that these conduits may improve durability compared to traditional homografts, although long-term outcomes are still being evaluated.

Despite the relatively high RVOT reoperation rates, significant advances in percutaneous pulmonary valve replacement (PVR) have transformed the management of RVOT conduit dysfunction. These transcatheter techniques have allowed for less invasive reinterventions, effectively delaying the need for open-heart surgery. Recent analyses indicate that freedom from surgical RVOT reintervention at 15 years is approximately 63.5% [20], reflecting the impact of evolving interventional strategies in extending conduit lifespan and reducing the burden of repeat operations [40].

### 4.3. Summary of Long-Term Complications

While the Ross procedure provides excellent survival and quality of life benefits, late reinterventions remain an important consideration. The autograft generally demonstrates good long-term durability, with the primary failure mechanism being neoaortic root dilation, whereas RVOT conduits predominantly fail due to progressive stenosis. Surgical modifications such as reinforcement techniques have shown promise in reducing autograft failure, but they are not universally applied due to concerns about restricting growth in younger children. Similarly, oversizing strategies and decellularized homografts offer potential solutions for RVOT reconstruction but require further long-term validation.

The introduction of percutaneous PVR represents a major advancement in reducing the need for repeat surgical interventions. While reinterventions remain a reality for many patients, the Ross procedure offers distinct advantages over conventional AVR in children by preserving growth potential, avoiding anticoagulation, and maintaining near-physiological hemodynamics.

## 5. Advantages and Considerations of the Ross Procedure

As outlined above, the Ross procedure offers several distinct advantages over conventional AVR, rendering it the preferred option for children older than one year. One of the most compelling benefits is its excellent long-term survival, which based on current follow-up data, appears comparable to that of the general population. This allows patients to lead active lifestyles without physical limitations, a key advantage over mechanical valve replacements that often require activity restrictions [23].

A major factor contributing to these superior outcomes is the favorable hemodynamic profile of the pulmonary autograft compared to prosthetic valves. Several studies have demonstrated that transaortic gradients on echocardiography remain low at rest and during exercise in Ross patients, closely resembling those of healthy controls [5,6]. In contrast, patients who undergo non-Ross AVR often experience higher transvalvular gradients, particularly under physiological stress, which may impair cardiac function and exercise capacity. This near-physiological hemodynamic performance provides long-term benefits in maintaining ventricular function, cardiac output, and overall cardiovascular health.

In addition to these hemodynamic advantages, a unique and critical benefit of the Ross procedure is the growth potential of the autograft, which no other surgical option can offer. Unlike prosthetic valves, which remain fixed in size and may become mismatched as the child grows, the pulmonary autograft has the ability to adapt and expand in parallel with somatic growth. This feature is particularly beneficial in pediatric patients, who require a long-term solution that accommodates their natural development.

Another major advantage of the Ross procedure is its low incidence of infective endocarditis, with reported rates ranging from 0.00% to 1.68% per year, significantly lower than those observed with prosthetic valves [41]. The risk of endocarditis in mechanical and bioprosthetic valves increases over time, and a recent analysis from the Society of Thoracic Surgeons database identified prosthetic valve endocarditis as a major risk factor for mortality [41]. The native tissue characteristics of the autograft may contribute to its resistance to infection, making it a safer long-term option compared to artificial prostheses.

Furthermore, because the Ross procedure avoids the need for lifelong anticoagulation, it significantly reduces the risk of anticoagulation-related complications. In contrast, patients with mechanical valves require continuous anticoagulation therapy, which increases the likelihood bleeding complications (0.02–0.39% per year), while thromboembolic events remain a concern despite anticoagulation [14]. Studies comparing the Ross procedure to non-Ross AVR have reported a lower incidence of thromboembolism in Ross patients (0.9% vs. 2.4%) [23], further reinforcing its safety profile.

Despite these advantages, the Ross procedure is not the ideal first-line option for all pediatric patients, particularly neonates and infants under one year of age. Studies have consistently shown that reintervention rates are higher in this population, suggesting that the Ross procedure may not provide the same long-term benefits when performed at such an early stage of life. This is likely due to a combination of greater hemodynamic stress, technical challenges, and increased conduit failure rates in neonates.

## 6. Conclusions

The Ross procedure has emerged as the gold standard for AVR in children older than one year, offering superior survival, near-physiological hemodynamics, the ability to grow with the patient, a lower risk of infective endocarditis, and avoidance of lifelong anticoagulation. However, reinterventions remain common, affecting both the pulmonary autograft and RVOT conduit. While the autograft failure rate is relatively low, progressive neoaortic root dilation can necessitate reoperation in some. Meanwhile, RVOT conduits, which lack growth potential, are prone to deterioration over time. In neonates and infants, the role of the Ross procedure remains less defined, considering the inferior outcomes.

## 7. Future Directions

To refine patient selection and improve long-term outcomes, ongoing research is focused on optimizing surgical techniques, refining selection criteria, and evaluating alternative management strategies for the youngest patients. One promising approach involves using an initial aortic valvuloplasty as a bridge to delay the Ross procedure in carefully selected neonates and infants, allowing for more favorable conditions at the time of surgery. Studies have shown that when the Ross procedure is performed after a failed valvuloplasty, outcomes remain comparable to those of primary Ross procedures [42]. This approach is supported by a recent meta-analysis demonstrating satisfactory outcomes with aortic valve repair and suggesting that reintervention can often be postponed for several years [43]. Moreover, a comparative study of aortic valve repair versus the Ross procedure reinforces this strategy, reporting better overall survival with valve repair but a higher likelihood of reintervention [44]. Other techniques, such as the Ozaki procedure, have shown promise in adults; however, recent data suggest less favorable outcomes in children when compared to the Ross procedure [45]. Additionally, advancements in percutaneous valve technologies hold the potential to extend the durability of the RVOT conduit and reduce the frequency of surgical reinterventions. International collaborative efforts are underway to generate high-quality data on long-term outcomes to help optimize surgical techniques and improve patient management.

## Data Availability

No new data were created or analyzed in this study. Data sharing is not applicable to this article.
